# Exercise Training and Beta-Alanine-Induced Muscle Carnosine Loading

**DOI:** 10.3389/fnut.2015.00013

**Published:** 2015-05-07

**Authors:** Tine Bex, Weiliang Chung, Audrey Baguet, Eric Achten, Wim Derave

**Affiliations:** ^1^Department of Movement and Sports Sciences, Ghent University, Ghent, Belgium; ^2^Department of Radiology, Ghent Institute for Functional and Metabolic Imaging, Ghent University, Ghent, Belgium

**Keywords:** beta-alanine, muscle contractions, sport supplements, carnosine loading, training

## Abstract

**Purpose:**

Beta-alanine (BA) supplementation has been shown to augment muscle carnosine concentration, thereby promoting high-intensity (HI) exercise performance. Trained muscles of athletes have a higher increase in carnosine concentration after BA supplementation compared to untrained muscles, but it remains to be determined whether this is due to an accumulation of acute exercise effects or to chronic adaptations from prior training. The aim of the present study was to investigate whether high-volume (HV) and/or HI exercise can improve BA-induced carnosine loading in untrained subjects.

**Methods:**

All participants (*n* = 28) were supplemented with 6.4 g/day of BA for 23 days. The subjects were allocated to a control group, HV, or HI training group. During the BA supplementation period, the training groups performed nine exercise sessions, consisting of either 75–90 min continuous cycling at 35–45% W_max_ (HV) or 3 to 5 repeats of 30 s cycling at 165% W_max_ with 4 min recovery (HI). Carnosine content was measured in soleus and gastrocnemius medialis by proton magnetic resonance spectroscopy.

**Results:**

There was no difference in absolute increase in carnosine content between the groups in soleus and gastrocnemius muscle. For the average muscle carnosine content, a higher absolute increase was found in HV (+2.95 mM; *P* = 0.046) and HI (+3.26 mM; *P* = 0.028) group compared to the control group (+1.91 mM). However, there was no additional difference between the HV and HI training group.

**Conclusion:**

HV and HI exercise training showed no significant difference on BA-induced muscle carnosine loading in soleus and gastrocnemius muscle. It can be suggested that there can be a small cumulative effect of exercise on BA supplementation efficiency, although differences did not reach significance on individual muscle level.

## Introduction

When a nutritional supplement is ingested with the aim to raise its concentration and accumulation in skeletal muscle cells, it is necessary to understand what factors are controlling the myocellular uptake and storage of that molecule. Literature on creatine supplementation shows that muscle creatine loading is enhanced when muscle activity is increased during creatine ingestion (1, 2). Harris et al. (1) showed a higher increase in creatine content in a trained leg compared to an untrained leg after supplementation combined with acute exercise, while Robinson et al. (2) showed that a single bout of exhaustive exercise before creatine supplementation can already markedly augment muscle creatine accumulation. However, it is not clear whether the potentiating effect of contractile activity on muscle loading of nutritional supplements is specific to creatine only, or a more universal mechanism. In the latter case, it could also affect carnosine loading via beta-alanine (BA) supplementation.

In recent years, there has been an increasing interest in BA as nutritional supplement in athlete populations (3–5). Chronic BA supplementation (4–10 weeks) has consistently been shown to elevate muscle carnosine concentrations with 40–80% (6–8), which can be beneficial for high-intensity (HI) exercise performance (9, 10). Carnosine, a dipeptide of l-histidine and BA, occurs in high concentrations in human skeletal muscles (11). The ergogenic effect of elevated carnosine concentrations is possibly based on its functions as proton buffer (12), and calcium regulator (13), or a combination of these functions (14, 15) in skeletal muscle. BA is the rate-limiting precursor to increase muscle carnosine levels, and therefore supplementation with BA is the most effective way to increase muscle carnosine levels. Until now, the parameters to optimize the supplementation strategy (e.g., dose or timing of intake) are not fully understood and should be further investigated to result in clearer guidelines for athletes. Furthermore, there are large inter-individual differences in carnosine loading effectiveness that are hardly understood at present (16).

In recent years, only a few studies investigated the effect of exercise and training on muscle carnosine loading. Kendrick et al. (17) did not find a difference in a trained vs. untrained leg after 4 weeks of training combined with BA supplementation. However, in this study, training consisted of isokinetic training with limited training volume (10 × 10 maximal contractions, three to four training sessions per week, over 4 weeks). In contrast, Bex et al. (18) demonstrated that trained muscles (e.g., arm muscles of kayakers and leg muscles of cyclists) showed a higher accumulation of carnosine compared to the untrained muscles (e.g., leg muscles of kayakers and arm muscles of cyclists). Hence, both within and between athletes, trained muscles had approximately two-fold higher carnosine loading compared to untrained muscles for the same BA intake. This study used experienced athletes who trained at least 8 h per week in their specific sport, which suggests that exercise and/or training status appears to be a feasible determinant of muscle carnosine loading. As the study of Bex et al. (18) was observational rather than interventional, it was unclear whether these effects are due to either the acute response of exercise or the chronic adaptations of muscle induced by prior training, or a combination of both. The precise role of training intensity, duration, and volume on exercise-induced carnosine loading should be further revealed. Implementation of different acute exercise protocols can give a better insight on which of these various factors affect the effectiveness of the carnosine loading protocol.

It is already known that training by itself does not influence muscle carnosine content (17, 19–21), but the effect of exercise training on the increase in muscle carnosine content by BA supplementation is not yet clear. Therefore, the main purpose of this study is to investigate whether intramuscular carnosine loading following BA supplementation is influenced by training volume vs. training intensity.

## Materials and Methods

### Participants

Twenty-eight men volunteered to participate in this study. All gave their written informed consent and the study was approved by the local ethical committee (Ghent University Hospital, Ghent, Belgium). None of the subjects were vegetarian. All subjects were non-specifically trained, but some of them took part in some form of recreational exercise 1–3 times per week (jogging, cycling, etc.). None of the subjects were engaged in regular organized training. Subjects were divided in either a control group or training group. The control (non-training) group consisted of 10 subjects, who were asked to be inactive throughout the intervention period. The control group’s age, weight, and height were 22.0 ± 2.3 years, 72.4 ± 10.3 kg, and 178.0 ± 7.8 cm, respectively. The subjects in the training group were allocated to a high-volume (HV) (*n* = 9) or HI (*n* = 9) group, matched for age, weight, height, muscle carnosine concentration, VO_2max_ and maximal power output at the graded exercise test (W_max_). The age, weight, and height were 21.6 ± 1.5 years, 77.0 ± 7.6 kg, and 180.0 ± 4.0 cm for the HV group and 21.7 ± 2.1 years, 80.4 ± 14.9 kg, and 180.0 ± 6.0 cm for the HI group respectively.

The supplementation protocol lasted 23 days and involved ingesting 6.4 g/day (2 × 800 mg tablets, four times daily with at least 2 h apart) of slow-release BA (Carnosyn, Natural Alternatives International). The supplement batch tested negative for contamination from prohibited substances by an independent drug surveillance laboratory (HFL Sport Science, Cambridgeshire, UK). All subjects were advised to take the tablets together with meals, and the subjects in the training groups were also asked to take one of the doses just prior to their training sessions. None of the subjects reported side effects due to the supplementation. Muscle carnosine concentration was measured before and after supplementation by proton magnetic resonance spectroscopy (^1^H-MRS) in soleus and gastrocnemius medialis muscles in all subjects, and an incremental cycling test was performed in the training group before and after supplementation.

### Experimental protocol

#### Preliminary Incremental Cycling Test

Each subject in the training group performed a maximal ramp exercise test on an electrically braked cycling ergometer (Lode, Groningen, Netherlands). Oxygen consumption was measured continuously via a computerized breath-by-breath system (JaegerOxycon Pro, Hoechberg, Germany). Pedaling frequency was kept between 75 and 80 rpm. After a warm-up of 3 min at 50 W, the work load was increased by 35 W/min to the point the subjects failed to continue to pedal at 75 rpm. Maximal power output at the graded exercise test (W_max_) corresponded to the mean value achieved over the last 30 s of the incremental cycling test.

#### Training Protocol

The training protocol consisted of nine sessions spread over 21 days, with 1–2 days of recovery between the training sessions (Table 1). Both groups performed training on mondays, wednesdays, and fridays for 3 weeks. For the HI group, training consisted of repeated 30 s maximal cycling bouts at 165% of W_max_, interspersed with 4 min of recovery (cycling at 40–70 W at their preference). Training progression was implemented by increasing the number of repeats from three to five repetitions. For the HV group, training consisted of 75–90 min continuous cycling at an intensity corresponding to 35–45% of W_max_. Training progression in the HV group was implemented by increasing duration of exercise throughout the weeks.

**Table 1:** **Parameters of the high-volume (HV) and high-intensity (HI) training protocols**.

Parameter	HV	HI
Work intensity	35–45% of W_max_	165% of W_max_ (“all out”)
Exercise protocol (per session)	75–90 min of continuous exercise	3–5 repeats × 30 s ‘all out’
		4 min active recovery
Total training time per session	75–90 min	1.5–2.5 min (intervals only)
		18.5–27.5 min (including recovery)
Total training time over 3 weeks	743 min	18 min (intervals only)
		225 min (including recovery)

#### Determination of Muscle Carnosine Content

Before and after supplementation, the carnosine content was measured in soleus and gastrocnemius medialis muscles by proton magnetic resonance spectroscopy (^1^H-MRS), as previously described by Baguet et al. (22). As seen in the study of Bex et al (18), the plantar flexors showed a higher increase in carnosine content in the cyclists. For this reason, the calf muscles were chosen over quadriceps muscles. The subjects were lying in supine position, and the lower leg was fixed in a spherical knee coil. All the measurements were performed on a 3-T whole body MRI scanner (Siemens Trio, Erlangen, Germany). Single voxel point-resolved spectroscopy (PRESS) sequence with the following parameters was used: repetition time (TR) = 2,000 ms, echo time (TE) = 30 ms, number of excitations = 128, 1,024 data points, spectral bandwidth of 1,200 Hz, and a total acquisition time of 4.24 min. The average voxel size for the soleus and gastrocnemius medialis muscles was 40 mm × 12 mm × 30 mm and 40 mm × 12 mm × 30 mm, respectively. Following shimming procedures, the linewidth of the water signal was on average 25.1 and 26.7 Hz for soleus and gastrocnemius medialis muscles, respectively. The absolute carnosine content was calculated, as described before by Baguet et al. (22). A variation coefficient for repeated measurements within the same day (23) was 4.3% (soleus), 7.6% (gastrocnemius), and 4.7% (average of soleus and gastrocnemius), while the biological variability within a 6-week period (6) was 9.8% (soleus), 14.2% (gastrocnemius), and 9.5% (average of soleus and gastrocnemius).

#### Statistics

A 3 × 2 general linear model repeated measures ANOVA was performed to evaluate the muscle carnosine content after BA supplementation, with “intervention group” (control, HV and HI) as between-subjects factor and “time” (pre vs. post) as a within-subjects factor. In case of a significant interaction effect, a 2 × 2 general linear model repeated measures ANOVA was performed between the different intervention groups. A 2 × 2 general linear model repeated measures ANOVA was performed to evaluate the changes in performance data, with “training group” (HV and HI) as between-subjects factor and “time” (pre vs. post) as a within-subjects factor. Pearson correlation was calculated between baseline carnosine content and absolute increase in muscle carnosine. Pearson correlation was also used to see if changes in muscle carnosine content correlated with changes in W_max_ or VO_2max_. All analyses were done with SPSS statistical software (SPSS 21, Chicago, IL). All values are reported as mean ± SD and statistical significance was set at *P* < 0.05. Trends were being identified with statistical significance set at *P* between 0.05 and 0.10.

## Results

### Muscle carnosine loading

**Figure 1:**
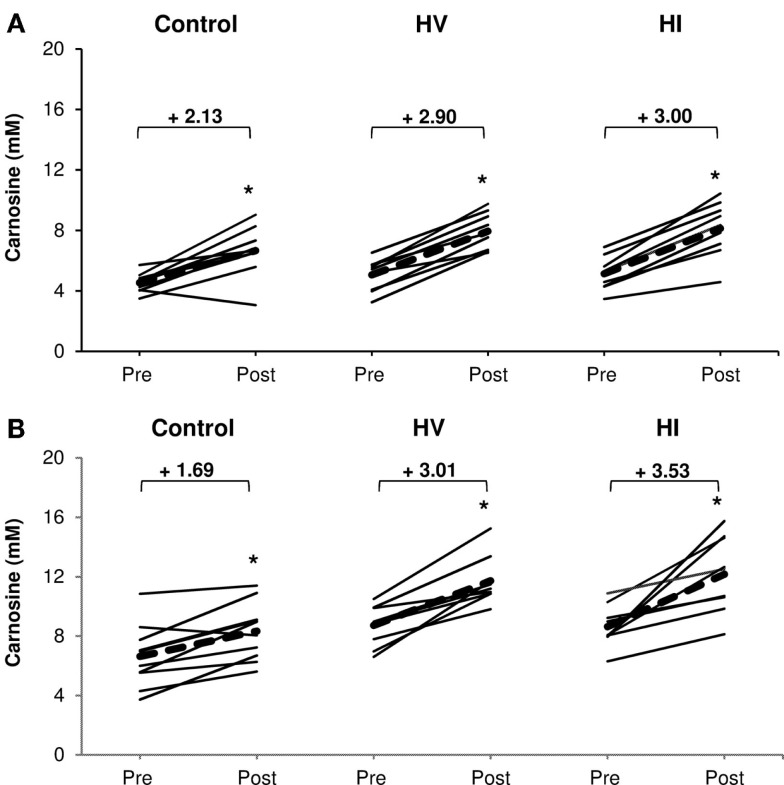
**Absolute increase in muscle carnosine concentration (mM) in soleus muscle (A) and gastrocnemius (B) in the control group, HV, and HI training groups**. Thin solid lines represent individual subjects and bold dashed lines represent group average. *P < 0.05 vs. pre.

**Figure 2:**
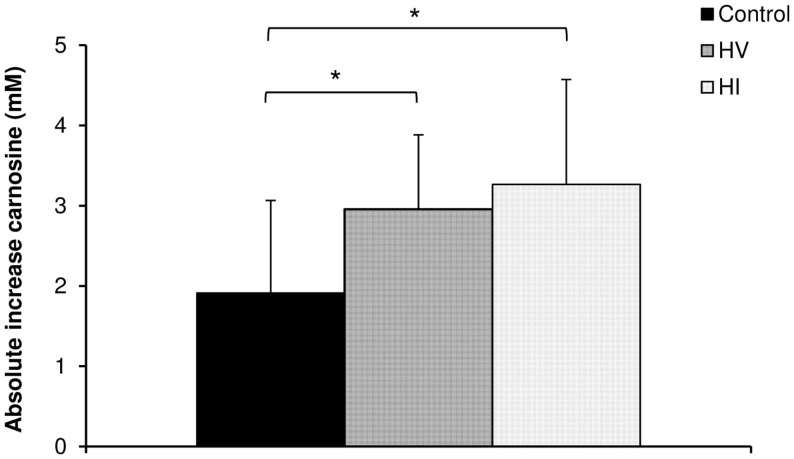
**Absolute increase in muscle carnosine concentration (mM) in the mean of the two muscles in the control group, HV, and HI training group**. *P < 0.05 vs. control group.

There was a significant increase in carnosine concentration in soleus, gastrocnemius, and mean of both muscles in response to BA supplementation in the control, HV, and HI training group (Table 2). Table 2 showed no significant time × group interaction effect in soleus (Figure 1A). In the gastrocnemius, a tendency to significant interaction effect (*P* = 0.080) was visible, and in the mean of the two muscles, a significant interaction effect (*P* = 0.039) was found. For gastrocnemius, there was a higher increase in HV group (+3.01 mM; *P* = 0.044) and HI group (+3.53 mM; *P* = 0.052) compared to the control group (+1.69 mM) (Figure 1B). Similar results were found in the mean of the two muscles, namely a higher absolute increase in the HV group (+2.95 mM; *P* = 0.046) and HI group (+3.26 mM; *P* = 0.028), compared to the control group (+1.91 mM) (Figure 2). The absolute increase in carnosine concentration in the HV and HI training group was not significantly different from each other, in neither gastrocnemius (*P* = 0.583) nor mean of the two muscles (*P* = 0.572).

**Table 2:** **Muscle carnosine content (mM) of the soleus, gastrocnemius, and mean of both muscles, pre and post supplementation**.

	Pre	Post	Interaction	Pre vs. Post
**M. Soleus**
Control group	4.54 (±0.62)	6.67 (±1.60)	0.201	<0.001
HV group	5.06 (±1.05)	7.95 (±1.21)	
HI group	5.13 (±1.09)	8.13 (±1.80)	
**M. Gastrocnemius**
Control group	6.63 (±2.10)	8.33 (±1.92)	0.080	<0.001
HV group	8.71 (±1.35)	11.72 (±1.63)	
HI group	8.64 (±1.38)	12.17 (±2.55)	
**Mean of both muscles**
Control group	5.59 (±1.25)	7.50 (±1.57)	0.039	<0.001
HV group	6.88 (±1.15)	9.84 (±1.32)	
HI group	6.89 (±0.93)	10.15 (±1.88)	

*Values are means (±SD) of 10 subjects in the control group, 9 subjects in the high-volume (HV), and 9 subjects in the high-intensity (HI) training group. Data are means ± SD*.

There was no correlation between baseline carnosine content and the absolute increase in muscle carnosine in soleus muscle (*P* = 0.493; *r* = 0.17) and gastrocnemius muscle (*P* = 0.386; *r* = -0.22), following BA supplementation. Furthermore, changes in muscle carnosine content were not correlated with changes in W_max_ (soleus: *P* = 0.831; *r* = 0.06, gastrocnemius: *P* = 0.207; *r* = 0.32) or VO_2max_ (soleus: *P* = 0.771; *r* = -0.07, gastrocnemius: *P* = 0.326; *r* = 0.25).

### Incremental VO_2max_ test

Following training, W_max_ was improved during the incremental VO_2max_ test with 2.2% in the HV group and 4.5% in the HI group (main effect of time, *P* < 0.01) (pre: 46.6 ± 7.6 vs. post: 49.5 ± 9.8 ml/min/kg), but there was no difference between the groups. VO_2max_ increased after training with 7.0% in the HV group and 5.0% in the HI group (main effect of time, *P* < 0.05) (pre: 369.8 ± 46.1 vs. post: 381.1 ± 38.8 W), with no difference between the groups. No effect of training on the differences in maximal heart rate was found in both groups (pre: 193.5 ± 8.2 vs. post: 191.2 ± 9.0 bpm).

## Discussion

The main purpose of this study was to investigate whether training volume or training intensity can enhance the efficacy of BA supplementation toward muscle carnosine loading. The potential effects of exercise on muscle metabolite loading could be in accordance with creatine, another nutritional supplement (1, 2). Both one bout of exercise (2) and 1 h training per day for 7 days (1) resulted in higher creatine loading in trained legs, compared to untrained legs following creatine supplementation. For BA, the role of exercise on muscle carnosine loading needs further clarification, as there is inconsistency between the results of Kendrick et al. (17) and Bex et al. (18). The current interventional study aimed to resolve the equivocal results and tried to get a better understanding of muscle carnosine loading effectiveness. Concerning the increase in carnosine concentration in this study, a higher absolute increase was found in both HV and HI group compared to the control group for the average muscle carnosine. These results confirm the data of Bex et al. (18), although the increases were smaller in current study. The effects in the study of Bex et al. (18) could also be partly the result of prior training status, namely training-induced increases in capillary density and higher expression of transporters and enzymes. As the current study conducted a training intervention in untrained subjects, the effect of prior training status was eliminated. Possibly, an additive effect of exercise on trained muscles causes the highest BA supplementation efficiency. This would implicate that trained athletes who train during BA supplementation have more pronounced muscle carnosine loading than untrained subjects who train.

The current study implemented two different training protocols to take a closer look at the exercise modalities and the inherent possible underlying mechanisms involved in exercise potentiation of muscle carnosine loading. The HV and HI training protocols used in the current design were inspired by the study of Gibala et al. (24). This study demonstrated that these training protocols cause similar effects on training-induced increases in muscle oxidative capacity, buffering capacity, and glycogen content. Given the large difference in training volume, their data demonstrated that HV and HI are comparable to induce rapid adaptations in skeletal muscle and exercise performance. The results of the VO_2max_ test in current study suggest that indeed both groups had a small but similar response on the different training sessions.

One group (HV group) performed continuous endurance training during the training period, while training in the HI group consisted of repeated 30 s maximal exercise bouts. The rationale for making these groups is based on some possible hypotheses. One likely explanation for exercise-induced carnosine loading would be the activity-related increase in blood flow and capillary recruitment, leading to higher interstitial BA concentrations, which are then available for transarcolemmal transport and intracellular storage. This mechanism is likely more sensitive to exercise duration and could have been an explanation if effects would have been more pronounced in HV than HI exercise. Yet, other mechanisms may be involved, such as the translocation and recruitment of transporters (TauT and PAT1) to take up BA in the myocytes in response to reactive oxygen species and/or contractile signaling (25). This would be in accordance with the glucose (GLUT4), creatine (CRT), and fatty acid transporter (FAT/CD36), which are recruited to the sarcolemma by a contraction stimulus (26–28). It is expected that this recruitment is more sensitive to exercise intensity, and therefore more intensely activated by HI exercise. The results of this study showed an equal increase in muscle carnosine concentrations in both training groups, suggesting that none of the above possibilities can be excluded at present. At physiological level, the precise mechanism to optimize BA-induced carnosine loading requires further investigation. It is possible that a combination of the above described or yet other mechanisms are responsible for enhancing the BA supplementation efficiency. Further research should include muscle biopsies to get a better insight in the mechanisms of exercise on carnosine loading efficiency. The advantage of muscle biopsies relates to the analysis on pooled single fiber level, which could determine the effect of the training stimulus in a muscle. In this study, ^1^H-MRS-based carnosine quantification is used instead of muscle biopsies, because it is a non-invasive technique that has a good repeatability in untrained (6) and trained (22, 29) humans. Furthermore, a greater part of the muscle is analyzed compared to muscle biopsies (30). However, from a practical viewpoint, these results can be translated into better guidelines for athletes, because both the HV and HI exercise stimuli seem to enhance the efficiency of the BA supplementation protocol.

Despite revealing new strategies to increase the efficiency of the BA supplementation protocol, the carnosine loading effectiveness remains low. Stegen et al. (31) were the first to calculate BA supplementation efficiency by dividing the molar increase in muscle carnosine by the total ingested molar amount of BA. Only 2.80% of ingested BA is actually incorporated into muscle carnosine (assuming that 40% of body mass is muscle mass). Recently, the study of Stegen et al. (31) showed that meal co-ingestion was able to increase the efficiency of BA supplementation, while Bex et al. (18) revealed that exercise and/or training status had an additional effect on the loading efficiency (up to 5.82% in trained muscles) (18, 31). In the current study, the loading efficiency of the HV and HI group was 5.17 and 5.67%, while it was 3.49% in the control group. Further research on BA metabolism is needed to get an understanding of the metabolic fate of the majority of the ingested BA.

Furthermore, this study confirms the high inter-individual variability in response to BA supplementation, with some subject displaying a supplementation-induced doubling of muscle carnosine content, yet with also two rare cases of non-responders (one in soleus and one in gastrocnemius of the control group). In our experience of the 109 subjects that have been supplemented chronically with BA in the various studies of our lab (6, 16, 18, 29, 31), only one did not show an increase in soleus carnosine content and only three did not show gastrocnemius carnosine loading. Even though this number of “non-responders” is rather small compared to other supplements (e.g., creatine), one could wonder why some subjects do not respond. The current study aimed to identify a key factor that influences inter-individual variability in muscle carnosine loading efficiency. Given the high remaining inter-individual variability, we conclude that there must be other elements, in addition to training (which was controlled in this study), which define responsiveness to BA supplementation. Future studies on the pharmacokinetics and metabolism of BA could shed light on this issue.

In summary, this study showed no significant difference on muscle carnosine loading after HV and HI exercise training. However, a beneficial effect on the efficiency of the muscular BA uptake and carnosine accumulation may occur, although this was not statistically confirmed on soleus and gastrocnemius muscles separately.

## Conflict of Interest Statement

The authors declare that the research was conducted in the absence of any commercial or financial relationships that could be construed as a potential conflict of interest.
